# Simulation-Based Approach to the Matching of a Dielectric-Filled Circular Waveguide Aperture

**DOI:** 10.3390/s24030841

**Published:** 2024-01-28

**Authors:** Songyuan Xu, Jiwon Heo, Byoung-Kwon Ahn, Chan-Soo Lee, Bierng-Chearl Ahn

**Affiliations:** 1 School of Electric and Computer Engineering, Chungbuk National University, Cheongju 28644, Republic of Korea; 2021197002@chungbuk.ac.kr (S.X.); heo1234@chungbuk.ac.kr (J.H.); 2 School of Autonomous Vehicle System Engineering, Chungnam National University, Yuseong-gu, Daejeon 34134, Republic of Korea; bkahn@cnu.ac.kr

**Keywords:** impedance matching, waveguide aperture, dielectric filled, open-end radiator

## Abstract

The circular waveguide aperture or open-end radiator, one of the canonical antenna elements, can be filled with a dielectric material for miniaturization. With dielectric filling, the aperture reflection increases and impedance matching is necessary. This paper presents a simple but innovative simulation-based approach to the aperture matching of a dielectric-filled circular waveguide aperture. By properly loading the aperture with two- or three-section dielectric rings, the impedance matching is possible over a wide frequency range starting slightly above the TE_11_-mode cutoff and continuing upward. The material for the aperture matching is the same as that filling the waveguide. The proposed matching structure is analyzed and optimized using a simulation tool for the dielectric constant *ε_r_* of the filling material ranging from 1.8 to 10. For *ε_r_* ≥ 5, the unmatched reflection coefficient ranges from −6.0 dB to −0.9 dB while the matched reflection coefficient is from −20.4 dB to −10.0 dB. The impedance matching has been achieved over more than an octave bandwidth.

## 1. Introduction

The circular waveguide aperture or open-end radiator is useful as an element in phased arrays [1,2] and as a reflector antenna feed [3,4]. Waveguides can be filled with a low-loss dielectric material for miniaturization [5,6], for protection from the environment [7,8], for feeding a dielectric rod antenna [9] and for some other purposes. Dielectric-filled waveguides can be used in the realization of antennas for radar sensors. For example, in [8], Xu and co-workers presented a dielectric-filled waveguide antenna element for a 3D imaging radar operating in high-temperature and excessive dust conditions.

In filling a waveguide with a dielectric material, it is often advantageous to use materials of different dielectric constants (*ε_r_*) for different applications. The operating frequency can be lowered for a given waveguide size, or the waveguide size can be reduced for a given frequency by a factor of 1/√*ε_r_*.

Traditionally, the Rexolite 1422^TM^, a cross-linked polystyrene plastic (with dielectric constant *ε_r_* = 2.54 and loss tangent tan*δ* = 0.00066 at 10 GHz [10]), and the Teflon^TM^ (*ε_r_* = 2.02 and tan*δ* = 0.0006 [11]) have been employed in dielectric-filled waveguides and in dielectric rod antennas. For *ε_r_* < 2, Eccostock LoK^TM^, a low-loss and low-weight thermosetting plastic by Laird Technologies (*ε_r_* = 1.7 and tan*δ* = 0.004) can be employed [12]. For *ε_r_* from 3 up to 30, Eccostock^®^ HIK500F, a series of low-loss, high-temperature, adjusted-dielectric-constant materials, can be used [13]. Table 1 shows the physical properties of the Eccostock^®^ LoK and Eccostock^®^ HIK500F. In the Eccostock^®^ HIK500F series, dielectric rods with *ε_r_* of 3, 4, 5, 6, 7, 8, 9, 10, 11, 12, 16, 20, 25 and 30 are available with tan*δ* < 0.002. On special orders, Laird Technologies supplies materials of other dielectric constants, e.g., 7.5. Table 2 lists a sampling of commercial dielectric materials with dielectric constants ranging from 1.7 to 250.0 and loss tangents from 0.0002 to 0.005.

The aperture reflection coefficient of a dielectric-filled waveguide aperture increases rapidly with increasing dielectric constant of the filling material, making the aperture matching necessary. A simple approach to the aperture matching of a dielectric-filled waveguide aperture is to extend the dielectric material a little distance beyond the aperture [4,6,21]. The shaping of the extended dielectric material reduces the reflection to some degree [6,8]. Other existing methods include a groove in the dielectric [21], an inductive iris [22], an airgap or low-k insert [22] and high-k low-k insert [23,24]. In previous works, the design goal has been the aperture matching for a specific frequency range and thus those works do not show broadband performance.

For some applications, it is necessary to make a dielectric-filled circular waveguide aperture work over a broad frequency range. Examples include broadband phased arrays and wideband reflector antennas. For bandwidth extension, it is to necessary to make a circular waveguide aperture operate from slightly above the first or fundamental TE_11_-mode cutoff (*f_c_*_TE11_) to slightly below the third TM_11_-mode cutoff (*f_c_*_TM11_). The second TM_01_ mode with cutoff at 1.66*f_c_*_TE11_ can be suppressed by using symmetrical structures in the circular waveguide. Since *f_c_*_TM11_/*f_c_*_TE11_ is 2.08, a bandwidth close to 2:1 is possible with a circular waveguide aperture.

In this paper, we present a simple but innovative technique for the broadband matching of a dielectric-filled circular waveguide aperture. A special emphasis is placed on making the lower operating frequency limit or the start frequency of the aperture as close to the TE_11_-mode cutoff as possible. The smaller the start frequency, the larger the size reduction ratio and the wider the bandwidth.

The proposed method is validated with simulation by CST Studio Suite^TM^ V2023, a widely used simulation tool in the antenna and RF community. We believe that the accuracy of this simulation tool is good enough to prove the proposed technique. All the dimensions of the proposed designs are given, and anyone can verify the results presented in this paper. To present experimental data, we need to design a high-performance coaxial-to-circular waveguide transition with a ratio bandwidth in excess of 2:1. The transition should work in a dielectric-filled circular waveguide. This is clearly another topic for research.

We acknowledge that there are certain design problems where the simulation-based design alone is not enough and an experimental verification is necessary. Examples may include structures that require new and critical fabrication methods and devices for which the dimensional accuracy is critical to the performance. We can find many papers where the design method is validated with simulation alone [24,25,26,27,28,29,30,31,32,33,34]. In our opinion, the aperture matching structure proposed in this paper is not about such a problem. In the next section, we will show the proposed technique with simulation-based design examples.

## 2. New Innovative Aperture-Matching Method

**Figure 1:**
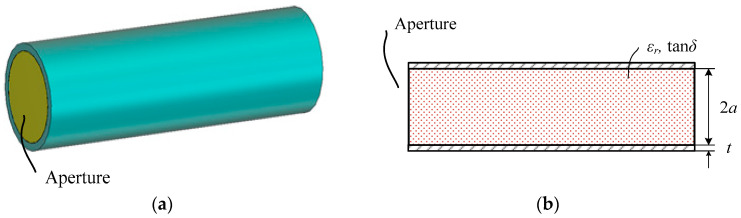
Dielectric-filled circular waveguide aperture (**a**) and its dimensional parameters (**b**).

Figure 1 shows an unmatched circular waveguide aperture. The waveguide inner wall diameter is 2*a* and the wall thickness is *t*. The waveguide is filled with a low-loss material with dielectric constant *ε_r_* and loss tangent tan*δ*. The dielectric filling lowers the operating frequency or reduces the waveguide diameter for a given operating frequency. For high size-reduction ratios, the dielectric constant of the filling material needs to be high, and this will greatly increase the reflection at the aperture.

**Figure 2:**
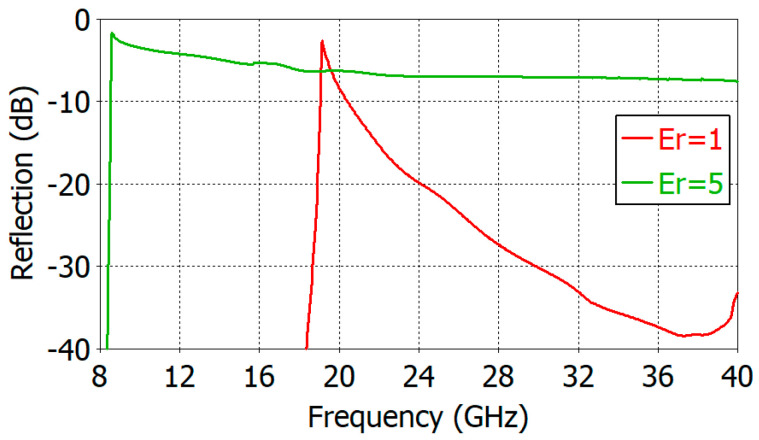
Reflection coefficient increase in the open end of a circular waveguide due to dielectric filling (*ε_r_* = 1 and 5.0, 2*a* = 9.20 mm, *t* = 0.80 mm).

Figure 2 shows the reflection coefficient of the apertures of an unfilled circular waveguide and of a circular waveguide filled with a material of *ε_r_* = 5.0. The waveguide diameter 2*a* is 9.20 mm and the wall thickness *t* is 0.80 mm. In Figure 2, one can observe a dramatic increase in the reflection coefficient when the waveguide is filled with a material of *ε_r_* = 5.0. In an unfilled circular waveguide, the dominant TE_11_-mode cutoff frequency (*f_c_*_TE11_) is 19.10 GHz, and the reflection coefficient reaches −10 dB at 20.43 GHz (1.070*f_c_*_TE11_) and a minimum value of −38.4 dB at 37.36 GHz. With *ε_r_* = 5.0, the dominant TE_11_-mode cutoff frequency (*f_c_*_TE11_) is 8.54 GHz and the reflection coefficient reaches −2.11 dB at 8.67 GHz (1.015*f_c_*_TE11_) and a plateau value of −6.9 dB at 22.48 GHz.

**Figure 3:**
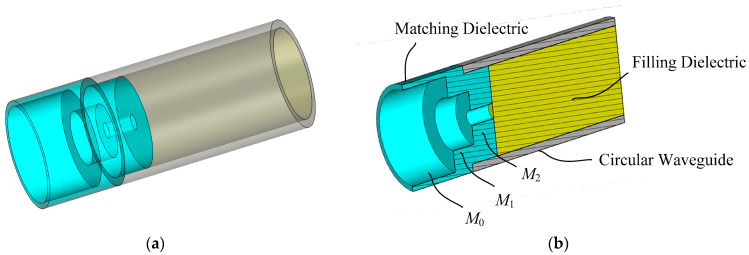
Aperture matching structure proposed in this paper: (**a**) transparent view and (**b**) cutaway view.

A simple and innovative method of the aperture matching proposed in this paper is depicted in Figure 3. A circular waveguide is filled with a dielectric material of dielectric constant *ε_r_* and loss tangent tan*δ* in the same way as in Figure 1. The same dielectric material as the filling material is placed in the waveguide open end for aperture matching. Broadband impedance matching is obtained by forming the matching material in a shape of two or three stacked rings which can be created by carving out concentric cylindrical volumes in the center and filling the carved-out space with air. Carving out the edge portion of a dielectric cylinder, i.e., solid coaxial dielectric cylinders of varying diameter, does not give a broadband aperture matching. More than three stacked rings can yield a wider bandwidth, but two or three rings give a bandwidth large enough for practical applications.

The matching structure can be thought of as a three-section (*M*_0_, *M*_1_ and *M*_2_) impedance transformer that converts the circular waveguide TE_11_-mode wave impedance into the free-space planewave impedance. The function of the matching rings is to gradually transform the effective dielectric constant of the medium from *ε_r_* of the dielectric-filled waveguide to 1 of the free space. The amount of the removed portion in the dielectric ring is increased as the wave propagates from the waveguide to the air, transforming the effective dielectric constant from *ε_r_* to 1. In this way, the guided TE_11_-mode wave in the circular waveguide is smoothly transformed to the TEM-mode planewave in the air. A broadband impedance matching is possible by proper dimensioning of the two or three concentric rings.

**Figure 4:**
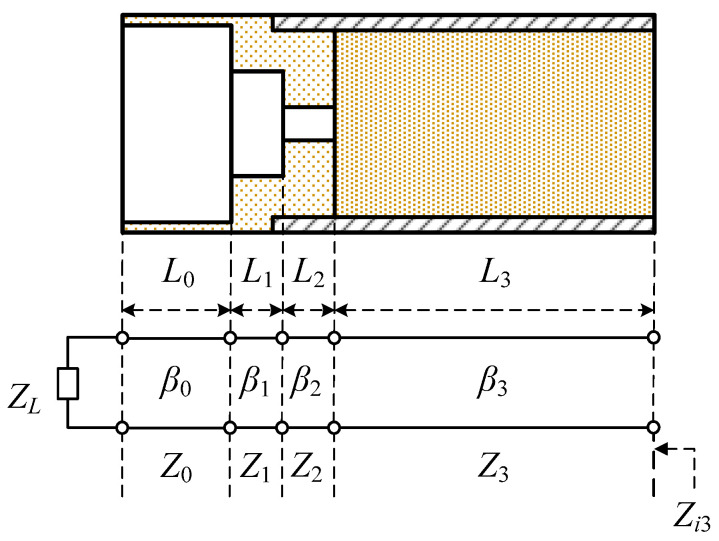
Equivalent circuit model of the proposed aperture matching structure.

Figure 4 shows a simplified equivalent circuit model of the proposed aperture matching structure. The parameter *Z_L_* is the characteristic impedance of the free space in front of the first matching element *M*_0_. The parameters *Z*_0_, *Z*_1_, *Z*_2_ and *β*_0_, *β*_1_, *β*_2_ and *L*_0_, *L*_1_, *L*_2_ are the characteristic impedance, propagation constant and length in the equivalent transmission line representation of the matching sections *M*_0_, *M*_1_ and *M*_2_, respectively. The parameters *Z*_3_, *β*_3_ and *L*_3_ are the characteristic impedance, propagation constant and length of the dielectric-filled circular waveguide, respectively. The parameter *Z_i_*_3_ is the input impedance at the circular waveguide port.

From the equivalent circuit model, the input reflection coefficient Γ at the circular waveguide port is obtained using

Γ = (*Z_i_*_3_ − *Z*_3_)/(*Z_i_*_3_ + *Z*_3_) (1)

The reflection coefficient Γ_0_ after the matching section *M*_0_ is given by

Γ_0_ = Γ*_L_*exp(−*j*2*β*_0_*L*_0_) (2)

where Γ*_L_* is the reflection coefficient at the input of *M*_0_ given by

Γ*_L_* = (*Z_L_* − *Z*_0_)/(*Z_L_* + *Z*_0_) (3)

The impedance *Z_i_*_0_ at the input of the second matching element *M*_1_ is obtained from Γ_0_ using

*Z_i_*_0_ = *Z*_0_ (1 + Γ_0_)/(1 − Γ_0_) (4)

By repeatedly applying Equations (2)–(4), we calculate *Z_i_*_1_, *Z_i_*_2_, and *Z_i_*_3_, from which we obtain the input reflection coefficient Γ given by (1).

The equivalent circuit model of Figure 4 will give accurate results if true values of the model parameters are used and the dielectric junction effect is included. A rigorous evaluation of the model parameters and the junction effect is very complicated if not impossible. Firstly, for a non-TEM transmission line, the characteristic impedance can be defined using the *VI*-definition, or the *PV*-definition or the *PI*-definition [35]. One has to determine on a theoretical basis which definition to use. Secondly, a simple transmission line model is not good enough for the proposed matching structure due to the fringing field in the aperture. The field near the aperture does not conform to that of a uniform transmission line where the transverse distribution of the electric and magnetic fields is invariant along the direction of wave propagation. Thirdly, the discontinuity effect of the dielectric step junction is difficult to model due to the complicated field structure of the aperture. Therefore, it is better to use an equivalent circuit model for the elucidation of the operating principles and to employ the numerical simulation and optimization for the actual design of the proposed matching structure.

**Figure 5:**
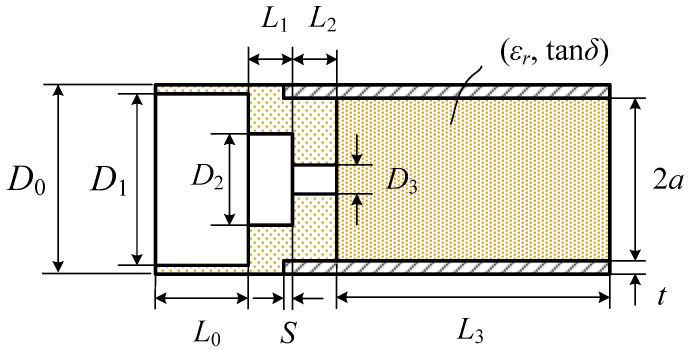
Dimensional parameters of the proposed aperture matching structure.

Figure 5 shows the dimensional parameters of the proposed matching structure. Fixed values of 9.20, 20.00 and 0.90 mm have been used for the inner wall diameter 2*a*, length *L*_3_ and wall thickness *t* of the circular waveguide. The parameter *S* is the position of the interface between the second (*M*_1_) and third (*M*_2_) matching sections relative to the end of the waveguide wall. It is positive if the *M*_1_-*M*_2_ interface is into the circular waveguide and negative otherwise. *D*_0_ is the diameter of the matching dielectric’s portion that is outside the waveguide. *D*_0_ is fixed at 2*a* + 2*t*. The remaining parameters are the hole diameters *D*_1_, *D*_2_, *D*_3_ and the lengths *L*_0_, *L*_1_ and *L*_2_ of the matching sections *M*_0_, *M*_1_ and *M*_2_, respectively.

The loss tangent (tan*δ*) of the filling and matching dielectric materials is fixed at 0.0008. A higher value of the loss tangent will not affect the aperture matching as far as it is small enough, for example, less than 0.008. It will, however, increase the attenuation of the waveguide. Power loss in a uniform transmission line is given by

*P* = *P*_0_ exp(−*βz*tan*δ*) (5)

where *z* is the propagation distance and *β* is the propagation constant of the waveguide TE_11_ mode. The power loss factor *P*/*P*_0_ of a one-wavelength long waveguide is 0.975 and 0.997 for tanδ of 0.008 and 0.0008, respectively. The impedance matching is controlled by the effective dielectric constant of the matching rings, which is in turn determined by the carved-out volume. Since the length of the impedance matching structure is not large—less than 1.35 times the waveguide diameter, as shown below in the design examples—the impedance matching performance is virtually the same for a loss tangent of 0.008 and 0.0008. This can easily be checked by simulation.

Starting from initial values *D*_1_ = 0.75(2*a*), *D*_2_ = 0.5(2*a*), *D*_3_ = 0.25(2*a*), *L*_0_ = *L*_1_ = *L*_2_ = 0.5(2*a*), *S* = 0, these parameters are optimized for *ε_r_* = 1.8, 2.5, 5.0, 7.5 and 10.0 using CST Studio Suite^TM^ V2023 for low reflection over as broad a frequency range as possible with the start frequency (reflection < −10 dB) as close as possible to the TE_11_-mode cutoff frequency of the dielectric-filled circular waveguide. For the two-section matching structure, the first matching section length *L*_0_ is set to zero.

**Figure 6:**
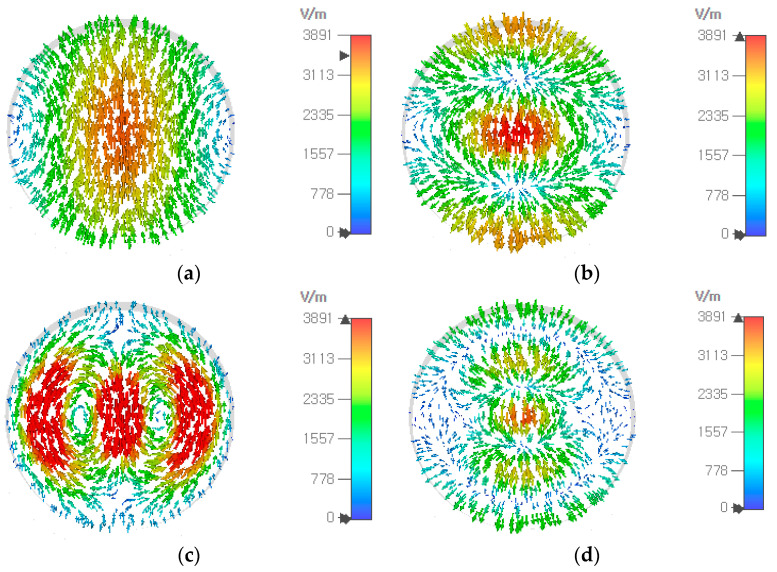
Electric field at 73 GHz in a circular waveguide (*ε_r_* = 1, diameter = 9.20 mm) of the fundamental TE_11_ (**a**), TM_11_ (**b**), TE_12_ (**c**) and TM_12_ (**d**) modes which are excitable along with the TE_11_ mode.

In designing an aperture-matched circular waveguide radiator, it is necessary to analyze the higher-order modes generated along with the fundamental TE_11_ mode. In a circular waveguide, the first 15 higher-order modes in the order of increasing cutoff frequencies are TE_11_, TM_01_, TE_21_, TE_01_/TM_11_, TE_31_, TM_21_, TE_41_, TE_12_, TM_02_, TM_31_, TE_51_, TE_22_ and TE_02_/TM_12_. The modes with the electric field being symmetric in the *E* and *H* planes, nonzero at the waveguide center and irrotational in the waveguide transverse plane, are easily excited when the fundamental TE_11_ mode is launched by a probe, a slot or other methods. They include TM_11_, TE_12_ and TM_12_ modes whose electric fields at 73 GHz have been obtained using CST Studio Suite^TM^ V2023 and are shown in Figure 6. The cutoff frequency of these modes in a 9.20-mm diameter waveguide is given in Table 3, including the *ε_r_* = 1 case.

By employing symmetric structures, it is possible to operate a circular-waveguide-based device from the TE_11_-mode cutoff to the TM_11_-mode cutoff. The cutoff frequency *f_c_*_,TM11_ of the TM_11_ mode is 2.08 times the fundamental TE_11_-mode cutoff frequency *f_c_*_,TE11_. Allowing for a guard band of 5% at *f_c_*_,TE11_ and *f_c_*_,TM11_, the frequency range will be from 1.05 *f_c_*_,TE11_ to 0.95 *f_c_*_,TM11_ (=1.98 *f_c_*_,TE11_) or a ratio bandwidth of 1.98/1.05 = 1.89. Since the level of the TM_11_-mode generation is dependent on a specific geometry of structures to be used in the waveguide, it is possible to operate a circular waveguide device above the TM_11_ mode cutoff. For example, Bang and Ahn have proposed a coaxial-to-circular waveguide transition operating with a ratio bandwidth of 2.18 [36].

**Figure 7:**
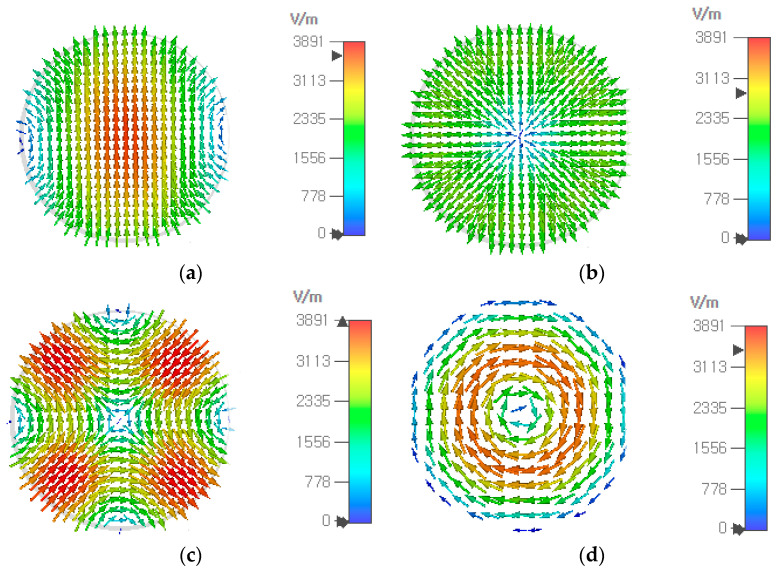
Electric field at 44 GHz in a circular waveguide (*ε_r_* = 1, diameter = 9.20 mm) of the fundamental TE_11_ (**a**), TM_01_ (**b**), TE_21_ (**c**) and TE_01_ (**d**) modes.

The modes with the electric field being antisymmetric in the *E* and *H* planes, or zero at the waveguide center, or rotational in the waveguide transverse plane, are not easily excited when the fundamental TE_11_ mode is launched, the first three of which are TM_01_, TE_21_ and TE_01_, whose cutoff frequencies are 1.31, 1.66 and 2.08 times the fundamental TE_11_-mode cutoff frequency, respectively. With *ε_r_* = 1 and 2*a* = 9.20 mm, they are 24.94, 31.68 and 39.74 GHz. Figure 7 shows the electric field of these three modes along with the TE_11_ mode at 44 GHz.

**Figure 8:**
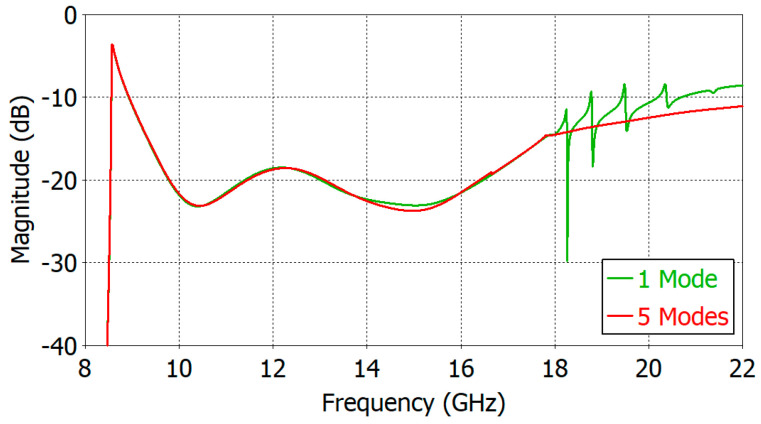
Effect of the higher-order mode inclusion on the reflection coefficient of a dielectric-filled circular waveguide aperture with *ε_r_* = 5.0.

In simulating a circular waveguide over a broad frequency range, the inclusion of the higher-order modes whose cutoff is in the frequency range of analysis has an effect on the reflection coefficient of the fundamental TE_11_ mode. Figure 8 shows the effect on the reflection coefficient of the higher-order modes in the frequency-domain analysis of a two-section matching structure with a filling material of *ε_r_* = 5.0. When only the TE_11_ fundamental mode is terminated properly (i.e., no reflection) but all higher-order modes are not included (i.e., total reflection), many resonance spikes appear due to multiple reflections of the higher-order modes between the aperture and the port plane. If we include five modes (TE_11_ mode + the first four higher-order modes TM_01_, TE_21_ and TE_01_/TM_11_), the spikes in the reflection coefficient will disappear. In the actual implementation of the proposed aperture matching structure, the termination condition of the higher-order modes depends on a device connected to the circular waveguide port.

In Figure 8, the resonance spikes occur at four frequencies; 18.27, 18.77, 19.48 and 20.36 GHz. The cutoff frequencies of the TM_01_, TE_21_, TE_01_/TM_11_, TE_31_ and TM_21_ modes in this waveguide are 11.16, 14.17, 17.77/17.77, 19.49 and 23.82 GHz, respectively. It is important to note that the resonance spikes occur at frequencies larger than the TM_11_ cutoff. In the design of the proposed matching structure, the frequency upper limit is set to the onset of the first resonance spike. The proposed matching structure can be used beyond the first resonance spike frequency if the generation of the TM_11_ mode is properly suppressed.

The optimization of the matching structure dimensions is performed in the following steps. The target frequency range for impedance matching optimization is set from the start frequency *f*_1_ = 1.01*f_c_*_,TE11_ to the end frequency *f*_2_ = 2*f_c_*_,TE11_, where *f_c_*_,TE11_ is the dominant TE_11_-mode cutoff frequency in the dielectric-filled circular waveguide given by

*f_c_*_,TE11_ = 299.792/(1.70629(2*a*)√*ε_r_*) (6)

In the first step of the design, a parametric analysis is carried out for the reflection coefficient versus dimensional parameters to find out the range of parameter values to be set in the optimization. The dimensions of the designed matching structure have been obtained using the ‘Trust Region Framework’ optimization algorithm provided by CST Studio Suite^TM^ V2023. The optimized design is typically obtained after 200 to 300 hundred iterations, which takes several hours on a desk-top computer.

Next, a first round of optimization is carried out for a target reflection coefficient of −10 dB, and optimum dimensions are obtained. With the dimensions obtained in the first round of optimization and the target reflection coefficient set at −15 dB, a second-round optimization is completed. Using the dimensions obtained in the second optimization round and with the reflection coefficient set at −20 dB, a third round of optimization is carried out. After the third round of optimization, good results are usually obtained.

**Figure 9:**
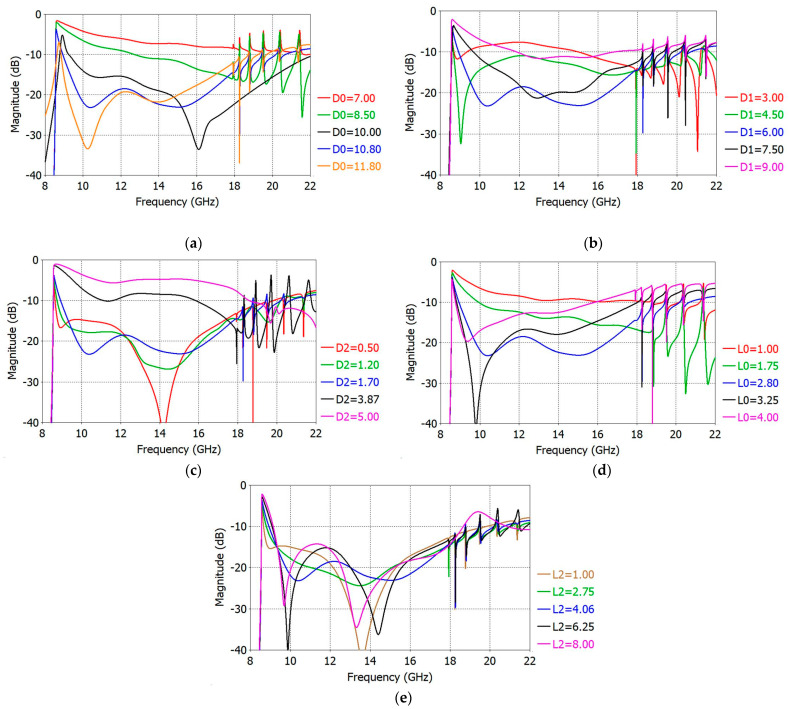
Reflection coefficient versus dimensional parameters at the aperture of a circular waveguide filled with material of *ε_r_* = 5.0 with (**a**) *D*_0_, (**b**) *D*_1_, (**c**) *D*_2_, (**d**) *L*_0_ and (**e**) *L*_2_ in mm.

Following the aforementioned procedures, we were successful in obtaining good aperture matching for *ε_r_* from 1.8 to 10. Figure 9 shows the reflection coefficient versus dimensional parameters of a two-section aperture matching design for *ε_r_* = 5.0. The curve in blue is for the reflection coefficient after optimization. In Figure 9, the spikes in the reflection coefficient at *f* > 18 GHz are caused by the exclusion of the higher-order modes in the waveguide port. As stated in the above, the resonance spikes are not generated if higher-order modes are included in the simulation. One design may require the lowest operating frequency with reflection < −10 dB and another design the lowest reflection at a specified frequency range. In Figure 9, one can see that this design flexibility is possible with a proper choice of dimensional parameters.

**Figure 10:**
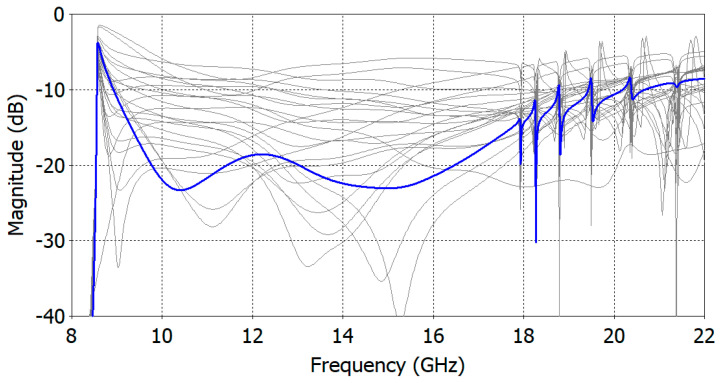
Changes in the reflection coefficient during an optimization of an aperture-matched dielectric-filled circular waveguide aperture with *ε_r_* = 5.0. The intermediate reflection coefficient curves are drawn in gray and the final one is in blue.

Figure 10 shows the change in the reflection coefficient during an optimization by CST Studio Suite^TM^ V2023. Starting from a high reflection coefficient, an optimization process finds the dimensions of the matching structure for a target reflection coefficient over a specified frequency range.

**Figure 11:**
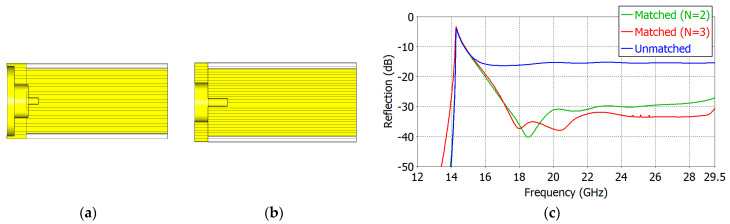
Three-section (*N* = 3) (**a**) and two-section (*N* = 2) (**b**) matching structures and the reflection coefficient (**c**) of the matched aperture with *ε_r_* = 1.8.

**Figure 12:**
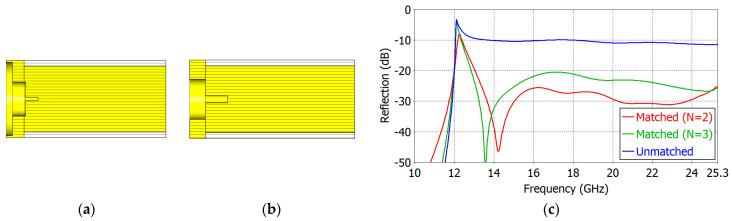
Three-section (*N* = 3) (**a**) and two-section (*N* = 3) (**b**) matching structures and the reflection coefficient (**c**) of the matched aperture with *ε_r_* = 2.5.

**Figure 13:**
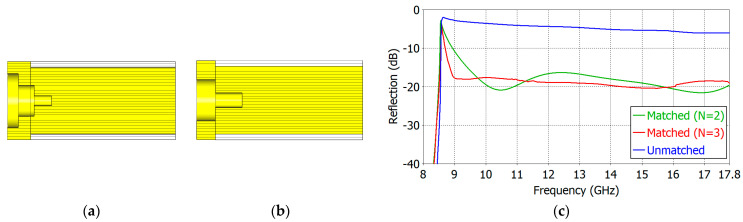
Three-section (*N* = 3) (**a**) and two-section (*N* = 2) (**b**) matching structures and the reflection coefficient (**c**) of the matched aperture with *ε_r_* = 5.0.

**Figure 14:**
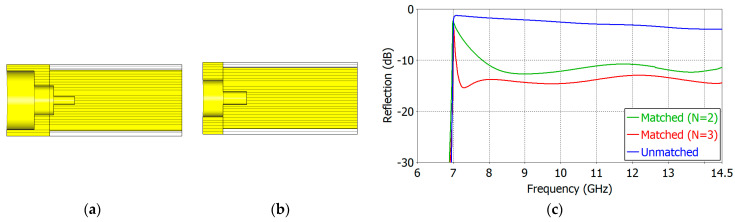
Three-section (*N* = 3) (**a**) and two-section (*N* = 2) (**b**) matching structures and the reflection coefficient (**c**) of the matched aperture with *ε_r_* = 7.5.

**Figure 15:**
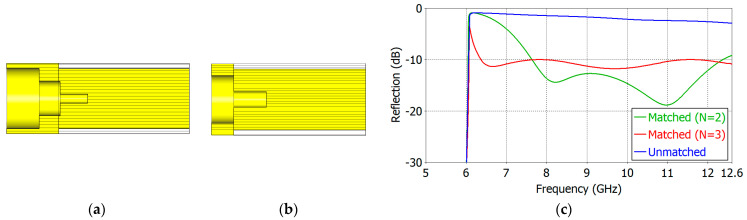
Three-section (*N* = 3) (**a**) and two-section (*N* = 2) (**b**) matching structures and the reflection coefficients (**c**) of the matched aperture with *ε_r_* = 10.0.

We have carried out aperture matching designs for *ε_r_* = 1.8, 2.5, 5.0, 7.5 and 10.0. We have made the lower limit of the operating frequency as close to the TE_11_-mode cutoff frequency as possible while maintaining a low reflection coefficient over a broad frequency range. Table 4 and Table 5 show the dimensions of the designed two- and three-section matching structures, respectively. The cross-section of the respective matching structure is also shown in Figure 11, Figure 12, Figure 13, Figure 14 and Figure 15.

Figure 11, Figure 12, Figure 13, Figure 14 and Figure 15 show the cross-section and the reflection coefficient of the aperture-matching structure with *ε_r_* = 1.8, 2.5, 5.0, 7.5 and 10.0, respectively. The frequency range in the figures is from a little below the TE_11_-mode cutoff to the onset of the spikes in the reflection coefficient. In all the cases, the reflection coefficient is dramatically reduced with the three-section (*N* = 3) matching as well as with the two-section (*N* = 2) matching. The three-section matching makes a starting frequency closer to the TE_11_-mode cutoff than the two-section matching and thus offers a wider bandwidth. The aperture matching can also be designed for a smaller reflection coefficient over a narrower frequency range.

Table 6 and Table 7 summarize the performance of the proposed aperture matching design with two- and three-section structures for *ε_r_* = 1.8, 2.5, 5.0, 7.5 and 10.0. The unmatched circular waveguide aperture has a reflection coefficient of less than −10 dB for *ε_r_* = 1.8 and 2.5. With dielectric constants of 5.0, 7.5 and 10.0, the unmatched reflection coefficient ranges from −6.0 to −0.9 dB, while with the proposed aperture matching the reflection coefficient is reduced to the −20 to −10 dB level.

**Figure 16:**
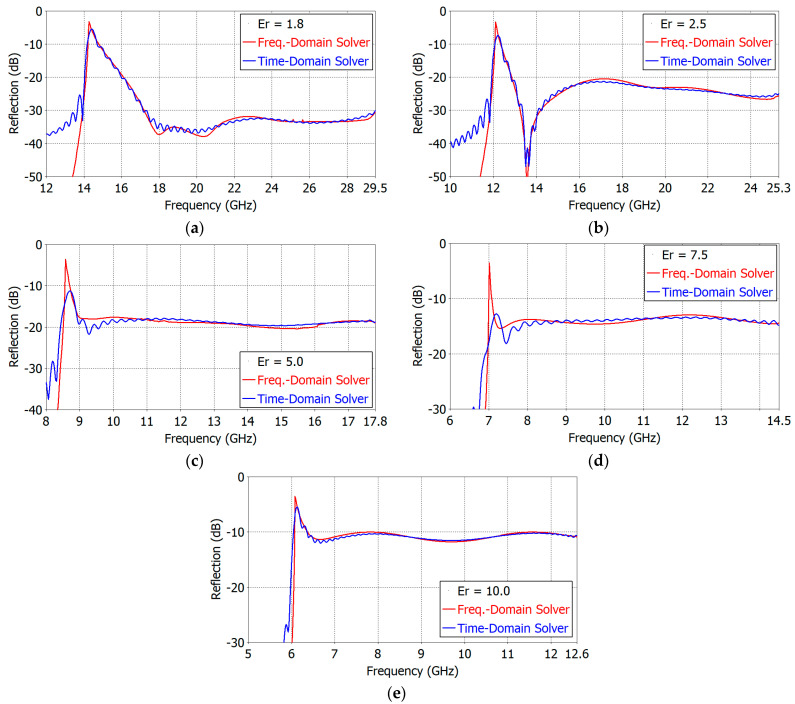
Reflection coefficients of the three-section aperture matching structure computed using the frequency- and time-domain solvers: (**a**) *ε_r_* = 1.8, (**b**) *ε_r_* = 2.5, (**c**) *ε_r_* = 5.0, (**d**) *ε_r_* = 7.5 and (**e**) *ε_r_* = 10.0.

In order to ascertain the simulation accuracy, we compared, in Figure 16, the reflection coefficients of the three-section matching structure computed using the frequency- and time-domain solvers for *ε_r_* from 1.8 to 10.0. The agreement between two simulation methods is excellent.

In Figure 11, Figure 12, Figure 13, Figure 14 and Figure 15, one can observe that the length of the matching structure is not large compared with the waveguide diameter. Table 8 and Table 9 summarize the matching structure lengths outside and inside the waveguide for the two- and three-section cases, respectively. The length of the outside portion ranges from 0.20 to 0.37 times the waveguide diameter (2*a*) in the two-section matching (*N* = 2) and from 0.30 to 0.87 times 2*a* in the three-section matching (*N* = 3), while the length of the inside portion ranges from 0.28 to 0.54 times 2*a* for *N* = 2 and from 0.19 to 0.48 times 2*a* for *N* = 3. The total length of the matching structure ranges from 0.48 to 0.91 times 2*a* for *N* = 2 and from 0.49 to 1.35 times 2*a* for *N* = 3. The compactness is one of the merits of the proposed matching structure.

Table 10 summarizes the impedance matching performance of the proposed three-section matching structures. As the dielectric constant *ε_r_* varies from 1.8 to 10.0, the reflection coefficient (|*S*_11_|) plateau increases from −31.7 dB to −10.0 dB. The ratio bandwidth for the reflection coefficient of less than the plateau reflection coefficient ranges from 1.68 to 2.01. A special merit of the proposed matching technique is that the start frequency (*f_S_*) for |*S*_11_| < −10 dB is very close to the TE_11_-mode cutoff, ranging from 1.015–1.051.

The reflection coefficient of the unmatched circular waveguide aperture at *f_S_* ranges from −10.7 dB to −0.93 dB for *ε_r_* from 1.8 to 10. If the aperture matching is carried out inside the waveguide to match an aperture with a reflection coefficient in the order of −3 dB to −1 dB, it will inevitably result in a very narrow bandwidth. In the proposed technique, however, the aperture matching starts outside the waveguide and ends inside the waveguide. The outside portion significantly reduces the aperture reflection, and a further reduction in reflection is carried out by the inside portion.

**Figure 17:**
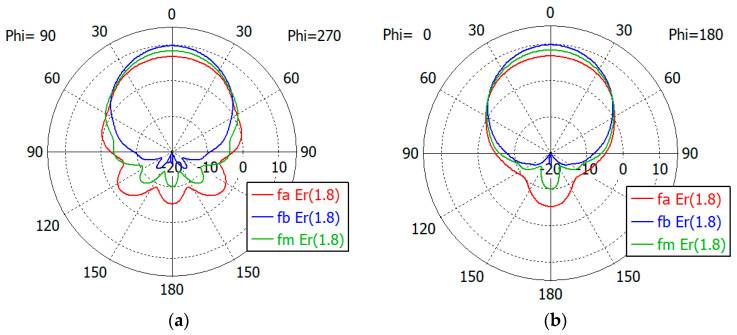
Gain patterns of a waveguide aperture with three-section matching (*ε_r_* = 1.8; *fa*, *f_m_*, *f_b_* = 17.5, 23.4, 29.4 GHz): (**a**) *E*-plane pattern and (**b**) *H*-plane pattern.

**Figure 18:**
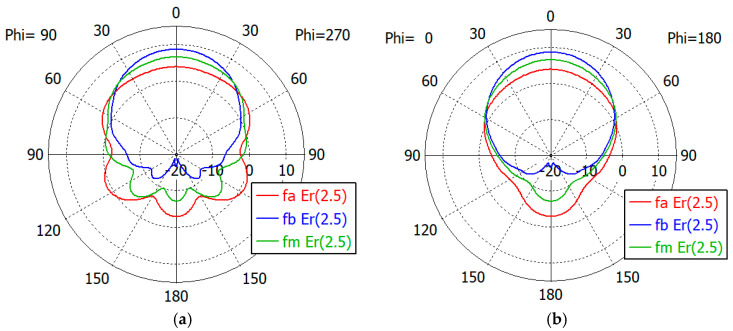
Gain patterns of a waveguide aperture with three-section matching (*ε_r_* = 2.5; *fa*, *f_m_*, *f_b_* = 12.9, 19.1, 25.3 GHz): (**a**) *E*-plane pattern and (**b**) *H*-plane pattern.

**Figure 19:**
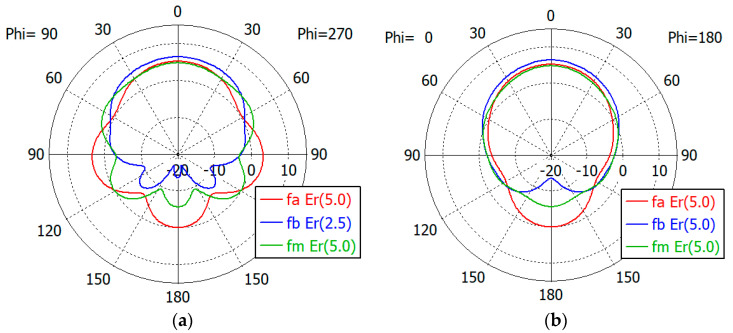
Gain patterns of a waveguide aperture with three-section matching (*ε_r_* = 5.0; *fa*, *f_m_*, *f_b_* = 8.9, 13.4, 17.8 GHz): (**a**) *E*-plane pattern and (**b**) *H*-plane pattern.

**Figure 20:**
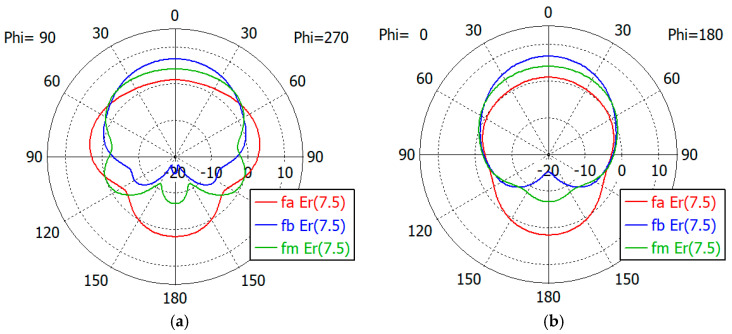
Gain patterns of a waveguide aperture with three-section matching (*ε_r_* = 7.5; *fa*, *f_m_*, *f_b_* = 7.2, 10.9, 14.5 GHz): (**a**) *E*-plane pattern and (**b**) *H*-plane pattern.

**Figure 21:**
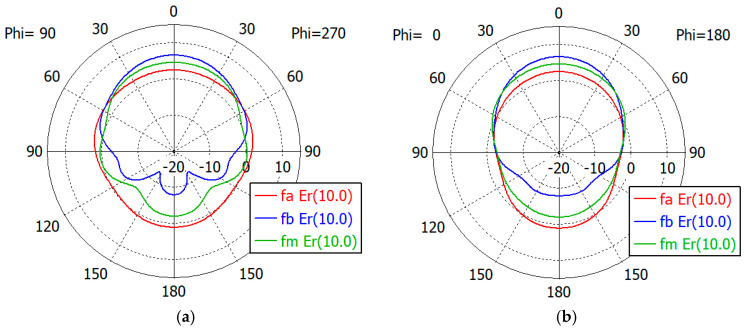
Gain patterns of a waveguide aperture with three-section matching (*ε_r_* = 10.0; *fa*, *f_m_*, *f_b_* = 6.4, 9.5, 12.6 GHz): (**a**) *E*-plane pattern and (**b**) *H*-plane pattern.

**Figure 22:**
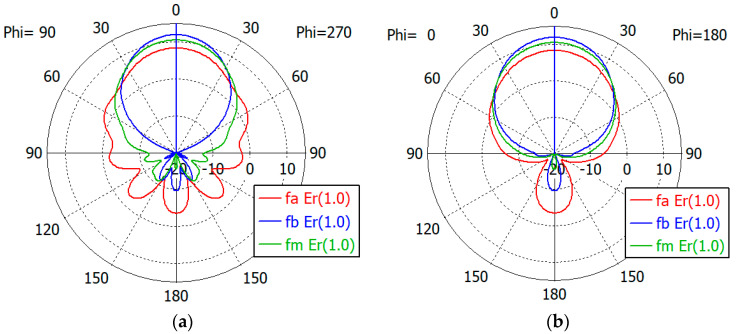
Gain patterns of a waveguide aperture with three-section matching (*ε_r_* = 1; *fa*, *f_m_*, *f_b_* = 20.4, 32.6, 40.0 GHz): (**a**) *E*-plane pattern and (**b**) *H*-plane pattern.

Figure 17, Figure 18, Figure 19, Figure 20 and Figure 21 show the *E*- and *H*-plane gain patterns of the designed matching structures with three-section matching, where *f_a_* is the start frequency for the reflection coefficient plateau and *f_m_* and *f_b_* are the middle and end frequencies of the plateau. For comparison, we present Figure 22 for an unfilled circular waveguide open end of the same diameter, where *f_a_* is the start frequency for the reflection coefficient < −10 dB, *f_m_* is the frequency for 2*a* = *λ* and *f_b_* is 40 GHz, an arbitrary upper frequency.

The aperture-matched dielectric-filled waveguide radiator shows gain patterns similar to those of an unfilled case. The maximum gain decreases as the dielectric constant increases, since the aperture dimension decreases compared to the wavelength in the air. For a given dielectric constant, the aperture’s gain increases as the frequency increases, except in the cases with *ε_r_* = 5.0, where the gain at *f_a_* is slightly larger than the gain at *f_m_*. At the start frequency, the gain in the backward direction (*θ* = 180°) is relatively large; −3.3 dBi with *ε_r_* = 1.0, and −5.0 dBi to 1.7 dBi with *ε_r_* from 1.8 to 10.0.

Table 11 summarizes the waveguide diameter 2*a* normalized by the wavelength in vacuum at the start frequency *f_a_* for the plateau reflection coefficient. Table 11 also shows the gain of the waveguide aperture at the start (*f_a_*), middle (*f_m_*) and end (*f_b_*) frequencies for the plateau reflection coefficient. The frequencies *f_a_* and *f_b_* are listed in Table 10. The aperture’s gain ranges from 6.7 dBi to 1.0 dBi at *f_a_*, from 10.1 dBi to 4.0 dBi at *f_m_* and from 12.1 dBi to 6.5 dBi at *f_b_* for *ε_r_* from 1.8 to 10. Table 11 also lists the gain of an air-filled waveguide aperture of the same diameter for comparison. The gain in this case is greater than that of the dielectric-filled aperture, since the operating frequency is higher. In Table 11, we note that with *ε_r_* = 10, the waveguide diameter is only 0.19 times the wavelength at *f_a_*, where the matched reflection coefficient is −10 dB, while the unmatched reflection coefficient is only −0.19 dB. We think that this is a remarkable result, which has not been achieved until now.

Table 12 compares the bandwidth performance of the proposed matching structures with previous works. First, we should mention that we could not compare the bandwidth under an equal condition since sufficient data have not been provided in previous works. In the proposed matching structures, the plateau reflection coefficient ranges from −31.7 dB to −13.1 dB for *ε_r_* from 1.8 to 7.5. If we use the condition |*S*_11_| < −10 dB in these cases, the bandwidth will be increased a little further. In [4], the frequency range for |*S*_11_| < −10 dB is not fully drawn. Therefore, bandwidth for |*S*_11_| < −20 dB is used in Table 12.

Even with the limited data provided in previous works, we can say that the level of bandwidth achievable with the proposed matching structure has not been heard of. In addition to the broadband aperture matching, the proposed method achieves |*S*_11_| < −10 dB very close to the cutoff frequency, maximizing the size reduction ratio. The proposed aperture matching structure is so simple that it can easily be machined or formed. For mechanical strength, the void air space in the matching rings can be filled with a material of low dielectric constant (1.03 ≤ *ε_r_* ≤ 1.30) with a subsequent adjustment of dimensional parameters.

## 3. Conclusions

A simple and innovative method has been presented for the broadband matching of a dielectric-filled circular waveguide aperture. By loading the aperture with two- or three-section dielectric rings, broadband impedance matching has been possible for the dielectric constant of the filling material ranging from 1.8 to 10.0. The dimensions of the matching structure have been obtained by computer-based optimization. The proposed matching structure is compact as well as geometrically simple so that it can easily machined or formed. With three-section matching rings, we have achieved a ratio bandwidth from 1.68 to 2.01 for a reflection coefficient ranging from –31.7 dB to –10.0 dB with a circular waveguide filled with a material with dielectric constants of 1.8, 2.5, 5.0, 7.5 and 10.0. With a dielectric constant greater than 5.0, the unmatched reflection coefficient ranging from −6.0 dB to −0.9 dB has been reduced to −20.4 dB to −10 dB. Start frequency for the reflection coefficient <–10 dB is very close to the TE_11_-mode cutoff, ranging from 1.015 to 1.051 times the TE_11_-mode cutoff frequency, which maximizes the size reduction ratio of the waveguide aperture. Existing methods of aperture matching of the dielectric-filled waveguide aperture do not provide a ratio bandwidth greater than 1.15. We expect that the proposed method would significantly contribute to the related art.

## Figures and Tables

**Table 1:** Physical properties of the Eccostock^®^ LoK and Eccostock^®^ HIK500F by Laird Technologies [12,13].

Typical Properties	Eccostock^®^ LoK		Typical Properties	Eccostock^®^ HIK500F
Temperature Range, °C (°F)	−70 to 150 (−94 to 302)		Temperature Range, °C (°F)	−56 to 204 (−69 to 400)
Frequency	60 Hz to 10 GHz		Density, g/cc	2.2
Density, g/cc	0.54		Dielectric Strength, Volts/mil	>300
Dielectric Constant	1.7		Dielec, Const. Accuracy, K < 16 (K > 16)	±3% (±10%)
Dielectric Strength, Volts/mil (kV/mm)	300 (11.8)		Dissipation Factor, 1 to 10 GHz	<0.002
Dissipation Factor	<0.004		Volume Resistivity, ohm-cm	>10^14^
Volume Resistivity, ohm-cm	10^14^		Flexural Strength, kg/cm^2^ (psi)	703 (10000)
Flexural Strength, kg/cm^2^ (psi)	420 (6000)		Coefficient of Linear Expansion, /°C	36 × 10^−6^
Coeff. of Linear Expansion, per °C (°F)	50 × 10^−6^ (28 × 10^−6^)		Izod Imp., kg-cm/cm (ft-lb/in)	1.65 (0.3)
Thermal Conductivity, W/mK	0.4		Outgassing, %TML (%CVCM)	0.47 (0.041)
Water absorption, %gain in 24 h at 25 °C	0.1			

**Table 2:** Commercial dielectric materials of varying dielectric constants.

Manufacturer	Product Name	Frequency (GHz)	Dielectric Constant	Loss Tangent Range
C-Lec Plastics [10]	Rexolite^®^ 1422	10	2.53	0.00066
Laird Technologies [12]	Eccostock^®^ LoK	10	1.7	<0.004
Laird Technologies [13]	Eccostock^®^ HIK500F	10	3, 4, 5, 6, 7, 8, 9, 10, 11, 12, 16, 20, 25, 30	<0.002
Laird Technologies [14]	Eccostock^®^ 0005	500	2.53	0.0005
Cuming Microwave [15]	C-STOCK^®^ 0005	-	2.54	0.0005
Laird Technologies [16]	Eccostock^®^ HIK	10	3, 3.5, 3.8, 4, 4.5, 5, 6, 7, 8, 9, 10, 11, 12, 13, 14, 15	<0.002
Cuming Microwave [17]	C-STOCK^®^ AK	-	3, 4, 5, 6, 7, 8, 9, 10, 12, 15, 20	<0.002
National Magnetics Group [18]	Microwave Dielectric Materials	9.4	4.3, 6.3, 9, 9.5, 12, 15, 16, 18, 20, 20, 25, 30, 50, 80, 100, 140, 160, 250	0.0002–0.005
Avient [19]	PREPERM^®^ Series	2.4	2.6, 2.7, 3.0, 3.2, 3.5, 4.0, 4.4, 5.0, 6.5, 8.0, 9.5, 10.0, 11.0, 12.0, 15.0, 23.0	0.0009–0.0045
Avient [20]	EDGETEK^®^ 7500 Series	1	3.0, 3.3, 3.4, 3.6, 3.8, 4.4, 4.8, 5.3, 5.9, 6.0, 7.0, 7.3, 9.0	0.0002–0.0012

**Table 3:** Cutoff frequencies of the modes excitable along with the TE_11_ mode in a dielectric-filled circular waveguide.

Matching Structure	Waveguide Diameter (mm)	*ε_r_*	*f*_cTE11_ (GHz)	*f_c_*_TM11_ (GHz)	*f_c_*_TE12_ (GHz)	*f_c_*_TM12_ (GHz)
0	9.20	1.0	19.10	39.74	55.30	72.77
1	9.20	1.8	14.23	29.62	41.22	54.24
2	9.20	2.5	12.08	25.14	34.98	46.02
3	9.20	5.0	8.54	17.77	24.73	32.54
4	9.20	7.5	6.97	14.51	20.19	26.57
5	9.20	10.0	6.04	12.56	17.49	23.01

**Table 4:** Dimensions of the two-section aperture matching structures (mm).

Matching Structure	*ε_r_*	*D*_0_	*D*_1_	*D*_2_	*D*_3_	*L*_0_	*L*_1_	*L*_2_	*L*_3_	*S*	2*a*	*t*
1	1.8	10.80	0	5.35	1.10	0	1.80	2.60	20.00	0	9.20	0.80
2	2.5	10.80	0	5.50	1.00	0	2.10	3.00	20.00	0	9.20	0.80
3	5.0	10.80	0	6.00	1.70	0	2.80	4.06	20.00	0	9.20	0.80
4	7.5	10.80	0	5.50	2.00	0	3.00	3.50	20.00	0	9.20	0.80
5	10.0	10.80	0	7.19	2.46	0	3.37	4.94	20.00	0	9.20	0.80

**Table 5:** Dimensions of the three-section aperture matching structures (mm).

Matching Structure	*ε_r_*	*D*_0_	*D*_1_	*D*_2_	*D*_3_	*L*_0_	*L*_1_	*L*_2_	*L*_3_	*S*	2*a*	*t*
1	1.8	9.80	9.97	4.73	0.94	1.05	1.97	1.70	20.00	0.27	9.20	0.80
2	2.5	9.80	10.28	4.74	0.52	0.89	1.88	1.73	20.00	0.24	9.20	0.80
3	5.0	9.80	7.48	4.31	1.38	1.51	2.19	2.89	20.00	0.49	9.20	0.80
4	7.5	9.80	8.90	4.47	1.20	0.79	3.36	2.93	20.00	0.58	9.20	0.80
5	10.0	9.80	9.29	5.26	1.39	5.01	3.21	4.22	20.00	0.21	9.20	0.80

**Table 6:** Reflection coefficient of the two-section matching structure (*N* = 2).

Matching Structure	*ε_r_*	Frequency Range (GHz)	Matched Reflection Coefficient (dB)	Unmatched Reflection Coefficient (dB)
1	1.8	16.8–29.5	−40.1 to −27.0	−16.3 to −15.4
2	2.5	13.1–25.3	−46.3 to −25.3	−11.4 to −9.5
3	5.0	9.6–17.8	−20.86 to −16.3	−6.0 to −3.3
4	7.5	7.9–14.5	−12.7 to −10.8	−4.0 to −1.7
5	10.0	7.6–12.4	−18.8 to −10.0	−2.8 to −1.4

**Table 7:** Length of the three-section matching structure (*N* = 3).

Matching Structure	*ε_r_*	Frequency Range (GHz)	Matched Reflection Coefficient (dB)	Unmatched Reflection Coefficient (dB)
1	1.8	17.2–29.5	−37.9 to −30.6	−16.3 to −15.4
2	2.5	12.9–25.3	−50.0 to −20.5	−11.4 to −9.2
3	5.0	9.0–17.8	−20.4 to −17.6	−6.0 to −2.8
4	7.5	7.1–14.5	−15.4 to −12.9	−1.3 to −4.0
5	10.0	6.4–12.6	−11.8 to −10.0	−2.9 to −0.9

**Table 8:** Length of the two-section matching structure (*N* = 2) normalized by the waveguide diameter.

Matching Structure	*ε_r_*	Length *L_A_* Outside the Waveguide (mm)	*L_A_*/(2*a*)	Length *L_B_* Inside the Waveguide (mm)	*L_B_*/(2*a*)	(*L_A_* + *L_B_*)/(2*a*)
1	1.8	1.80	0.20	2.60	0.28	0.48
2	2.5	2.10	0.23	3.00	0.33	0.56
3	5.0	2.56	0.28	3.60	0.39	0.67
4	7.5	3.00	0.33	3.50	0.38	0.71
5	10.0	3.37	0.37	4.94	0.54	0.91

**Table 9:** Length of the three-section matching structure (*N* = 3) normalized by the waveguide diameter.

Matching Structure	*ε_r_*	Length *L_A_* Outside the Waveguide (mm)	*L_A_*/(2*a*)	Length *L_B_* Inside the Waveguide (mm)	*L_B_*/(2*a*)	(*L_A_* + *L_B_*)/(2*a*)
1	1.8	2.76	0.30	1.71	0.19	0.49
2	2.5	2.60	0.28	3.03	0.33	0.61
3	5.0	3.21	0.35	2.89	0.31	0.66
4	7.5	6.50	0.71	3.79	0.41	1.12
5	10.0	8.01	0.87	4.43	0.48	1.35

**Table 10:** Impedance matching performance of the proposed three-step aperture-matching structure.

Matching Structure	*ε_r_*	TE_11_−Mode Cutoff (*f*_cTE11_) (GHz)	Start Frequency (*f_S_*) for |*S*_11_| < −10 dB (GHz)	*f_S_*/*f_c_*_TE11_	|S_11_| at *f_S_* in Unmatched Case (dB)	Plateau Value of |*S*_11_| (dB)	Frequency Range (*f_a_*−*f_b_*) for Plateau |*S*_11_| (GHz)
1	1.8	14.23	14.83	1.042	−10.7	−31.7	17.5–29.4
2	2.5	12.08	12.32	1.020	−6.44	−20.5	12.9–25.3
3	5.0	8.54	8.67	1.015	−2.11	−18.8	8.9–17.8
4	7.5	6.97	7.09	1.017	−1.27	−13.1	7.2–14.5
5	10.0	6.04	6.35	1.051	−0.93	−10.0	6.4–12.6

**Table 11:** Gain of the aperture-matched dielectric-filled waveguide open end.

Matching Structure	*ε_r_*	Wavelength in Vacuum *λ*_0_ (mm) at *f_a_*	Waveguide Dia. In Wavelength (2*a*/*λ*_0_)	Gain (dBi) at *f_a_*	Gain (dBi) at Middle Freq. *f_m_*	Gain (dBi) at End Freq. *f_b_*
0	1.0	14.71	0.63	8.4	10.1	12.1
1	1.8	20.23	0.45	6.7	8.4	9.8
2	2.5	24.35	0.38	3.9	6.6	8.7
3	5.0	34.60	0.27	5.2	4.0	6.7
4	7.5	42.31	0.22	1.0	4.0	6.7
5	10.0	47.24	0.19	2.4	4.5	6.5

**Table 12:** Comparison with previous works.

Work	Matching Scheme	Dielectric Constant (*ε_r_*)	Frequency (GHz)	Reflection (dB)	Ratio Bandwidth	Complexity
[4]	Protruding Dielectric Cylinder	2.5	8.1–9.3	−20	1.15	Low
[22]	Groove	4.1	9.0–9.5	−10	1.06	Low
[23]	High-k-Low-k Insert	2.2/6.3	Not specified	−17	1.10	High
This Work	Three Dielectric Rings	1.8	17.5–29.4	−31.7	1.68	Medium
		2.5	12.9–25.3	–20.5	1.96	
		5.0	8.9–17.8	–18.8	2.00	
		7.5	7.2–14.5	–13.1	2.01	
		10.0	6.4–12.6	–10.0	1.97	

## Data Availability

The data presented in this study are available in this article.
